# Genetic variation in the Estonian population: pharmacogenomics study of adverse drug effects using electronic health records

**DOI:** 10.1038/s41431-018-0300-6

**Published:** 2018-11-12

**Authors:** Tõnis Tasa, Kristi Krebs, Mart Kals, Reedik Mägi, Volker M. Lauschke, Toomas Haller, Tarmo Puurand, Maido Remm, Tõnu Esko, Andres Metspalu, Jaak Vilo, Lili Milani

**Affiliations:** 1 Institute of Computer Science, University of Tartu, Tartu, 50409 Estonia; 2 Estonian Genome Center, Institute of Genomics, University of Tartu, Tartu, 51010 Estonia; 3 Department of Physiology and Pharmacology, Section of Pharmacogenetics, Karolinska Institutet, Stockholm, 171 77 Sweden; 4 Department of Bioinformatics, Institute of Molecular and Cell Biology, University of Tartu, Tartu, 51010 Estonia; 5 Science for Life Laboratory, Department of Medical Sciences, Uppsala University, Uppsala, 751 44 Sweden

**Keywords:** Population genetics, Medical genetics, Next-generation sequencing, Genome-wide association studies

## Abstract

Pharmacogenomics aims to tailor pharmacological treatment to each individual by considering associations between genetic polymorphisms and adverse drug effects (ADEs). With technological advances, pharmacogenomic research has evolved from candidate gene analyses to genome-wide association studies. Here, we integrate deep whole-genome sequencing (WGS) information with drug prescription and ADE data from Estonian electronic health record (EHR) databases to evaluate genome- and pharmacome-wide associations on an unprecedented scale. We leveraged WGS data of 2240 Estonian Biobank participants and imputed all single-nucleotide variants (SNVs) with allele counts over 2 for 13,986 genotyped participants. Overall, we identified 41 (10 novel) loss-of-function and 567 (134 novel) missense variants in 64 very important pharmacogenes. The majority of the detected variants were very rare with frequencies below 0.05%, and 6 of the novel loss-of-function and 99 of the missense variants were only detected as single alleles (allele count = 1). We also validated documented pharmacogenetic associations and detected new independent variants in known gene-drug pairs. Specifically, we found that *CTNNA3* was associated with myositis and myopathies among individuals taking nonsteroidal anti-inflammatory oxicams and replicated this finding in an extended cohort of 706 individuals. These findings illustrate that population-based WGS-coupled EHRs are a useful tool for biomarker discovery.

## Introduction

Variability in drug response constitutes a major public health concern, accounting for 2.5–10.6% of all hospital admissions [1]. Direct healthcare costs per case of hospitalization due to adverse drug effects (ADE) range from €943.40 to €7192.36 [2]. Around 30% of novel therapeutics will eventually be affected by ADEs that are not identified in clinical trials [3]. Genetic variations affecting the absorption, distribution, metabolism, excretion, and toxicity (ADMET) of drugs cause an estimated 20–30% of the variability in drug response between individuals [4]. Mechanistic associations between drug response and pharmacogenetic variants in genomic coding regions are well understood, but sparse functional information is available for noncoding regions, with studies failing to identify or replicate significant results [5]. Uncovering associations between pharmacogenes and drugs increasingly relies on large-scale initiatives that organize and produce knowledge of variants in different populations and highlight actionable variants that can be clinically implemented to improve health outcomes [6–8]. Electronically collected medical information on treatment courses, methods, and outcomes linked with genetic data is an invaluable resource for studies of genotype-phenotype relationships. However, studies in which these data sets are systematically integrated have been lacking.

Here, we applied the whole-genome sequencing (WGS) data of more than 2200 Estonian Biobank participants and imputed genotypes of more than 16,000 participants [9], as well as corresponding longitudinal drug prescription data and extensive electronic health records (EHRs) from sequenced individuals. Leveraging these data, we present a comprehensive hypothesis-free discovery study of genotype-drug response associations on a population scale [10].

## Materials and methods

### WGS variant calling, quality control, and genotype imputation

The 2284 WGS samples were sequenced at the Genomics Platform of the Broad Institute. Sequenced data were jointly variant-called and quality controlled as outlined in Supplementary Methods and in Guo et al. [11]. The resulting WGS data was used to construct the Estonian reference panel of 16.5 × 10^6^ SNVs [9]. This was used to impute genotypes of individuals genotyped at the Core Facility of the Estonian Genome Center with Infinium CoreExome-24 BeadChips (*n* = 6396), Illumina HumanCNV370-Duo BeadChips (*n* = 2658), or Illumina HumanOmniExpress Beadchips (*n* = 8138). Imputed variants were required to pass the WGS quality control, and have a call rate greater than 0.95 and minor allele count greater than 2. Summary level statistics of detected genetic variation have been submitted to dbSNP (build 152; accession number: 1063012), linked to BioProject (https://www.ncbi.nlm.nih.gov/bioproject; accession: PRJNA489787), and included among gnomAD data sets (r2.0.2, http://gnomad.broadinstitute.org). Additional details regarding imputation are provided in the Supplementary Methods.

### Electronic health records

Clinical information for Biobank participants was obtained from various EHR databases: Health Insurance Fund Treatment Bills (from 2004), Tartu University Hospital (from 2008), North Estonia Medical Center (from 2005) [10]. These were thereafter mined for multiple patient and drug prescription attributes as outlined in Supplementary Methods.

### Adverse drug effects

We used EHRs to assess ADE occurrence among study participants. To identify the diagnosed case as an ADE, we used a list of 79 ICD10 codes for possible drug-induced diagnoses and diagnoses described as “due to drugs” or “unspecified”. To confirm the association with drugs for ICD10 codes that did not have a direct relationship with the drug in the diagnosis description (e.g., Myositis, unspecified—M60.9), we manually searched the NDHRD medical records for affirmative comments from the treating physician about the link between the disease and the drug. This process was followed to examine possible ADE cases among 2240 Biobank participants who had WGS data (at the time of the study, medical records were not available for other participants). All ADEs that were self-reported by Biobank participants at the time of recruitment were included in the final list of possible ADE cases. For added insight, we regrouped the 79 codes of possible ADE diagnoses into 12 diagnostic groups according to the leading pathophysiological mechanism/process and the main affected organ/organ system (Supplementary Table 1).

### Targeted pharmacogenetic variation

We compiled a list of 64 pharmacogenes that have been shown to be important in drug responses, using the core gene list from PharmaADME [12] and very important pharmacogenes from Pharmacogenomics Knowledgebase [13] (Supplementary Table 2). Effects of all variants called within pharmacogenetic genes were annotated by VEP [14] and subsequently filtered (Supplementary Methods). The novelty of called SNVs in pharmacogenes was determined by VEP 84 (dbSNP144) annotations.

### Functionality of targeted pharmacogenetic variations

Definitions for LoF variants were adapted from MacArthur et al. [15]. We used annotations from VEP and the LOFTEE plugin of VEP to identify predicted stop-gain, frameshift, or essential splice-site variants, and excluded ancestral alleles and variants located in the last 5% of the transcript. All non-LoF variants, whose effects were predicted by VEP as moderate-to-high under the Sequence Ontology term, were classified as missense.

To define potential variation in promoter regions, we studied regulatory regions within 5000 base-pairs upstream of all pharmacogene 5’ ends. We used the UCSC Table Browser to extract Fasta-formatted reads for these regions, which we used as input for the prediction tool Match (v9.0) [16] to extract transcription factor binding sites. This tool uses the TransFac [17] transcription factor library for binding motifs. We only retained variants with HepG2 ChIP-seq data published by the HudsonAlpha Institute, Broad Institute, and Sydney Genomics Collaborative program made available through ENCODE [18].

### Validation of *CYP2D6* variant calls

The chromosome 22 portion of *CYP2D6 (NM_001025161.2)* is entirely located in a region annotated as a segmental duplication. We compared *CYP2D6* CNV estimates and k-mer counts of the corresponding region as a proxy for validating *CYP2D6* variant calls. Specifics of CNV and k-mer discovery / filtering have been outlined in Supplementary Methods.

### *CYP2D6* star allele and HLA-haplotype calling

To determine the *CYP2D6* star alleles, we used the Constellation tool (v0.5) [19] and all called variants within 5000 base-pairs up- and downstream of *CYP2D6*. Each individual was assigned a *CYP2D6* star allele haplotype and diplotype. For 6-digit-precision *HLA*-haplotype calling, we used the SNP2HLA tool [20] in the major histocompatibility complex region for individuals with available WGS data (*n* = 2240). Observed *HLA-B* haplotypes were tabulated with R software (v3.2.0) [21].

### Validation of known pharmacogenetic associations

We selected all drug/variant associations curated with high confidence in PharmGKB (level of evidence 1 A, 1B, 2 A 2B) [13] to test their relevance in the joint data set of WGS and genotyped samples. We tested all allele/variant and drug combinations with a logistic regression (LR) model, after excluding drug/variant combinations having fewer than 500 participants with associated drug prescriptions, genes lacking alternative variant carriers, and drugs without recorded ADE diagnoses among participants (Supplementary Methods).

For *CYP2D6* and *HLA-B* alleles, we used the allele estimates from Constellation and SNP2HLA. For all other multi-SNV alleles, an individual was assigned as an allele carrier if at least one allele variant was heterozygous or homozygous at a variable site. We again used a LR model to test the relationship between ADE occurrence with genotype among participants with drug prescription using the following co-variates: BMI, sex, age, four PCs, and genotyping platform (WGS or genotype chip). Analysis was performed in Plink v1.9 [22] with a nominal *p*-value threshold of 0.05.

### Effect of pharmacogenetic variation on ADE occurrence

To examine the role of pharmacogenomic variants (*n* = 1314) in PharmGKB gene-drug associations, we extracted associations from PharmGKB (level of evidence 1–4) and evaluated ADE occurrences among participants with prescriptions of drugs that had been associated with any variant in the tested pharmacogenomic variant’s gene. Genotypic effects of ADE prevalence differences among individuals with some drug prescriptions were tested with a LR model with the same co-variates as described in “Validation of known pharmacogenetic associations”. Variants that were missing from imputation panels were only tested based on WGS data. All associations with a *p*-value lower than 0.05 were then, if available, conditioned on all other significant gene variants reported in PharmGKB for tested gene-drug association. Co-occurrences of genetic variants, drugs, and ADEs were visualized as a Sankey flow diagram.

### Genome-wide association studies

We conducted a single-variant association analysis to identify, at the whole-genome level, variations that were associated with ADE occurrences among participants with specific drug prescriptions. Data from imputed genotyping assays and whole genomes were merged into a single VCF formatted file using bcftools. To obtain the optimal number of phenotypes and to increase association power, we grouped active pharmaceutic ingredients into subgroups of the fourth-level ATC classification system [23]. One subgroup of drugs was included in the GWAS analysis as one phenotype when drugs were prescribed to at least 1000 Biobank participants, resulting in selection of 43 phenotypes for analysis (Supplementary Table 3). For each phenotype, we included only participants that had drug prescriptions in the corresponding ATC group, and we studied the prevalence of ADE relative to the genotype. Analysis was performed with Plink (v1.9) on variants with an AF of at least 1% using an additive genetic logistic model. Associations were corrected for the same co-variates as in previous analyses.

### Variant selection for replication

After filtering GWAS results using a suggestive genome-wide significance level *p*-value threshold of <10^−6^, we evaluated remaining loci based on associated genes and phenotype (active pharmaceutic ingredient) using different sources of background information (Supplementary Table 4; sheet 1). Various databases were reviewed to evaluate biological plausibility of tentative variants (Supplementary Methods). All selected loci were visualized by LocusZoom plots with 1000 G data (v0.4.8, 03.2012, hg19 assembly) and LD information from the European population [24]. Variants were filtered for MAF greater than 5%.

### GWAS replication

As discussed in “ADEs”, all ADE incidences were regrouped into 12 diagnostic groups (Supplementary Table 1). To refine ADE phenotypes further, we reanalyzed significant associations in the GWAS with the LR model, defining participants with ADEs in a specific diagnostic group as cases and individuals without any ADEs as controls. At the time of the study, there were participants in the Estonian Biobank for whom no genotyping or sequencing data were available. Therefore, we were able to draw analysis samples from the same population as the discovery set to perform replication analysis in an independent data set from the Biobank (Supplementary Table 5). In this way, we ensured that the samples used in the replication analysis were broadly similar to those used in the initial study [25] (Supplementary Methods).

Three of the five replicated SNVs identified using methods from “Variant selection for replication”, were genotyped with predesigned TaqMan assays. For two SNVs, we genotyped different SNVs in LD because the regions that covered the SNVs were not suitable for Taqman assay design (Supplementary Table 4; sheet 2). Genotype effects were tested by an additive LR model, corrected by age, BMI, and sex. Replication results were significant if the independent Bonferroni correction *p*-value for five tests was less than 0.01. *p*-values for the meta-analyses of discovery and replication sets were obtained by using the sum-of-z method in the R package metap (v0.8) [26].

### Analysis of the *CTNNA3* locus

Several additional analyses were performed to investigate the unveiled association between c.1047+29179 T>C (rs75495219) in *CTNNA3 (NM_001127384.2)* with the occurrence of myopathy-related ADEs among individuals who had been prescribed oxicams. First, we tested for an association between SNV rs75495219 in 387 unique cases of myopathy/myositis regardless of drug intake to rule out variant association with muscle pain and inflammation. Next, we conditionally adjusted for variant c.1047+201065 C>G (rs61866214) that peaked (*p* = 1.3 × 10^−5^) in a previously tested rs75495219 association. We applied VEP to examine if any other *CTNNA3* gene variants in LD with rs75495219 are exonic or significantly affect gene function. Gene expression influences were examined through regulatory elements by using GTEx portal and RegulomeDB [27]. Properties of *CTNNA3* and oxicams were analyzed in the same way as described in “Variant selection for replication”. Interactions of *CTNNA3* with other genes were evaluated by using the ConsensusPathDB database [28].

## Results

By analyzing the WGS data of 2240 individuals from the Estonian Biobank, we identified 29.1 × 10^6^ novel variants. Most of these variants (73.1%) were rare (minor allele frequency [MAF] < 1%), with 18.6% of variants having an Estonian population MAF greater than 5%. To study clinically relevant variations in the sequenced genomes, we established a set of 1314 loss-of-function (LoF), missense, and putative high-impact variants in promoter regions of 64 candidate genes prominently involved in drug pharmacokinetics and pharmacodynamics (Supplementary Table 2) [13]. Of these variants, 12.5% were common (MAF ≥ 5%), 80.3% were rare (MAF < 1%), 42.6% were singletons, and 20.6% were novel (Table 1). The high proportion of rare variants in pharmacogenes indicates the need for sequencing-based approaches in studying pharmacogenetically important variation [29, 30]. Around 3% (*n* = 41) of ADMET variants were stop-gained or essential splice site (Supplementary File 1: Extended Table 1). Using the Variant Effect Predictor (VEP) tool, we annotated putative LoF variants in 25 of the 64 selected pharmacogenes, detected in 727 of the 2240 genomes from sequenced Biobank participants (Supplementary Table 6). In all, 58.5% of LoF variants were singletons or doubletons (MAF < 0.05%) (Supplementary File 1: Extended Table 2). Moreover, 32.5% of the participants carried at least one LoF variant in ADMET genes, with 3.5% of individuals being homozygous for at least one inactivated pharmacogene.

**Table 1:** Single-nucleotide variation (SNV) characteristics in whole-genome sequences from Estonian Biobank participants

(a) Variants in the Estonian Biobank discovery set	Whole genome		ADMET genes (*n* = 64)	
	*n*	%	*n*	%
Genes with variants	18,468		56	
Unique variants	29,108,287		1314	
Variant carriers	2240		2240	
Novel variants	11,508,281	39.5	267	20.3
Known variants	17,600,006	60.5	1047	79.7
MAF > 5%	5,403,215	18.6	164	12.5
1% ≤ MAF < 5%	2,444,670	8.4	95	7.2
0.5% ≤ MAF < 1 %	1,211,084	4.2	45	3.4
0.05% ≤ MAF < 0.5%	6,460,248	22.2	285	21.7
MAF < 0.05 %	13,589,070	46.7	725	55.2
AC = 1	10,617,607	36.5	560	42.6
AC = 2	2,971,463	10.2	165	12.6

(b)	Loss-of-function		Missense		Promoter region	
	*n*	%	*n*	%	*n*	%
Unique variants	41		567		706	
MAF > 5%	1	2.4	39	6.8	124	17.6
1% < MAF < 5%	0	0	38	6.7	57	8.1
0.5% < = MAF < 1 %	1	2.4	16	2.8	28	3.9
0.05% < = MAF < 0.5%	15	36.6	113	19.9	157	22.2
MAF < 0.05 %	24	58.5	361	63.6	340	48.1
AC = 1	21	51.2	279	49.2	260	36.8
AC = 2	3	7.3	82	14.5	80	11.3
Novel variants	10	24.3	134	23.6	123	17.4
Novel variants AC = 1	6	14.6	99	17.5	50	7.1
Known variants	31	75.6	433	76.4	583	82.5
Known variants AC = 1	15	36.6	180	31.7	210	29.7

*AC* allele count, *MAF* Minor allele frequency, *ADMET* absorption, distribution, metabolism, excretion, and toxicity, *n* number of variants

(a) Numbers and frequencies of detected variants in whole-genome sequences and targeted pharmacogenes

(b) Characterization of targeted pharmacogenetic variations in loss of function (LoF), missense, and regulatory (transcription factor binding sites in liver 5-kb upstream of gene start site) regions

Due to the complexity of the genome in these regions, we called variants of *HLA-B* and *CYP2D6* [31, 32] using specifically purposed calling tools [Constellation [19] and SNP2HLA [20]]. Highly polymorphic *HLA-B* exhibited 23 different alleles, with an allele frequency (AF) greater than 0.5% in 2,240 participants. The most frequently observed allele was HLA-B*07:02:01 with 15.6% (Supplementary File 1: Supplementary Figure 1). Detection frequency of the HLA-B*57:01:01 allele was 2.3%. This allele has been associated with abacavir-induced hypersensitivity reactions [33] and its frequency was within range of other European populations [34]. For *CYP2D6*, we used two independent methods for calling copy number variations (CNVs) within the gene. Copy numbers called with GenomeStrip [35] correlated well (*R*^2 = ^0.64) with results called by a k-mer-based approach (Supplementary File 1: Supplementary Figure 2). CNV analysis revealed that 4.93% of assessed Estonian individuals were heterozygous for the *CYP2D6* deletion allele CYP2D6*5, and one participant was homozygous.

**Fig. 1:**
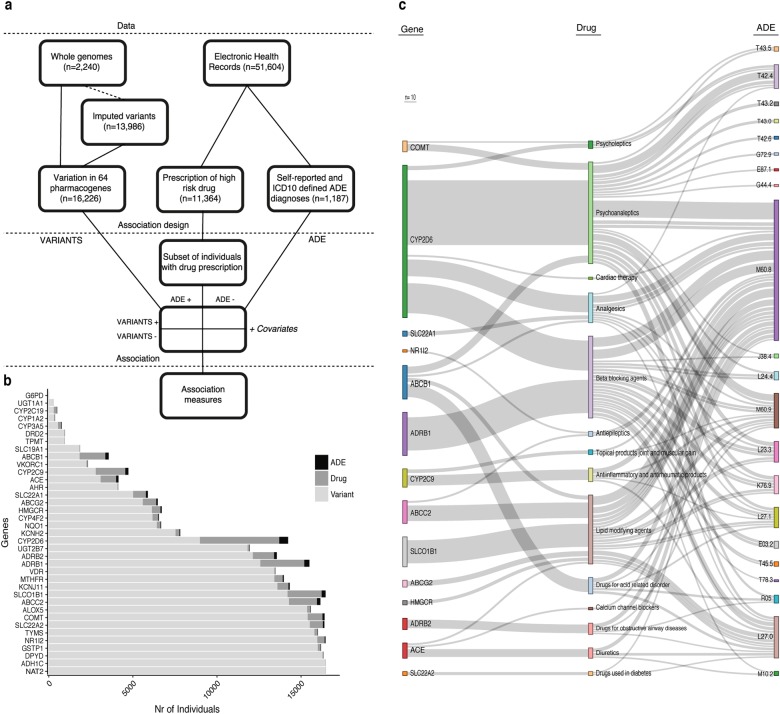
Overview of genetic variation, drug consumption, and adverse drug effect (ADE) data in electronic health records (EHRs). **a** Outline of pharmacogenomic variation, high-risk drug prescriptions, and ADEs. Drug prescriptions and medical histories in EHRs were combined with whole-genome sequencing data and imputed genotypes to investigate effects of genetic variation in 64 pharmacogenetically important genes on prevalence of ADEs among people with specific drug prescriptions. **b** Numbers of Estonian Biobank participants with variations in pharmacogenes (light gray bars), filled prescriptions of high-risk drugs with known genetic associations (dark gray bars), and diagnosed ADEs (black bars). **c** Flowchart visualizing co-occurrences of genetic variants, drug prescriptions, and ADEs among Estonian Biobank participants. Line thickness reflects the number of individuals with a given feature (minimum *n* = 10)

To explore the underlying genetics of ADEs, we overlapped data from national EHR databases with genetic variations of 64 highly pharmacogenetically relevant genes (Fig. 1). Within the period from January 2004 to August 2015 for which EHR data were available, 11,364 (70%) of the studied Biobank participants were prescribed drugs designated as high-risk for specific genotype carriers (“high-risk drug prescriptions”). Among them, 7997 individuals (70.3%) had putative high-impact polymorphisms in genes associated with the prescribed drugs.

We extracted ADEs from EHRs using a list of 79 ICD10 codes combined with self-reported incidences of adverse effects (Supplementary Table 1). ADEs ranged from very specific (drug-induced allergic dermatitis, ICD10 code: L23.3) to broader and less certain definitions (Myositis, unspecified, ICD10 code: M60.9) [36]. The discovery set of 16,226 Biobank participants included 1187 individuals with possible ADE diagnoses. The top 20 most common ADEs identified among participants are listed in Extended Table 3 (Supplementary File 1). Overall, 805 Biobank participants showed (i) putative high-impact polymorphisms in 56 of the 64 pharmacogenes, (ii) were prescribed at least one drug associated with the polymorphic gene, and (iii) experienced at least one ADE (Fig. 1).

To validate our approach of combining population scale sequencing data with EHR information, we set out to test 337 previously described high-confidence associations in the selected 64 pharmacogenes (Supplementary Table 7). Many associations could not be tested, due to absence of the respective variant in the Estonian cohort (*n* = 74), missing drug prescription information (*n* = 129), no known ADE diagnosis (*n* = 16), or missing variant carriers among individuals with the drug prescription (*n* = 18). For statistical power considerations, we excluded all associations for which we could interrogate fewer than 500 individuals (*n* = 63) [37]. Importantly, we were able to replicate high-confidence relationships between the CYP2D6*6 allele and ADEs related to tramadol (*p* = 0.035; odds ratio [OR] = 2.67) and amitriptyline (*p* = 0.02; OR = 6.0) (Table 2).

**Table 2:** Validation of previously reported PharmGKB associations in 64 pharmacogenes

						ADE/drug		No ADE/drug				
Gene	Rs number/star allele (variants in LD)	Variant HGVS term (hg19)	Drug (ATC code)	AA	Level of evidence	Variant carriers	Non-carriers	Variant carriers	Non-carriers	OR (95% CI)	*p*-value	Post-conditional *p*-value
(a) Significant variant/drug associations												
*CYP2D6*	CYP2D6 *6	chr22:g.42525086_42525086delA	Amitriptyline (N06AA09)	*6	1 A	5	77	5	532	6.0 (1.33–29.28)	0.020	NA
*CYP2D6*	CYP2D6 *6	chr22:g.42525086_42525086delA	Tramadol (N02AX02)	*6	1B	9	182	16	1181	2.7 (1.04–6.53)	0.035	NA
*ABCB1*	rs2032582	chr7:g.87160618 A > T	Atorvastatin (C10AA05)	T	3	3	27	2	126	6.9 (1.09–43.1)	0.037	NA
*ADRB1*	rs1801252	chr10: g.115804036 A > G	Verapamil (C08DA01)	G	3	11	8	49	129	3.6 (1.36–9.46)	0.018	NA
*SLC22A1*	rs12208357	chr6::g.160543148 C > T	Tramadol (N02AX02)	T	3	52	139	236	961	1.5 (1.07–2.15)	0.009	NA
*CYP2D6*	rs3892097	chr22::g.42524947 C > T	Sertraline (N06AB06)	T	3	12	55	102	194	0.4(0.102–0.74)	0.016	NA
(b) Significant gene/drug associations												
*ABCC2*	rs7910642	chr10:g.101541579 G > A	Simvastatin (C10AA01)	A	3	9	55	138	366	0.4 (0.2–0.9)	0.0181	0.0121
*ABCC2*	rs45441199	chr10: g.101591737 T > C	Carbamazepine (N03AF01)	C	3	3	33	3	256	7.7 (1.5–39.5)	0.0112	0.0177
*ACE*	rs4980 (rs4358)	chr17:g.61574642 G > A (chr17:g.61571907 G > A)	Torasemide (C03CA04)	A	3	7	100	13	635	3.4 (1.3–8.8)	0.0243	0.0243
*COMT*	rs56104268	chr22:g.19946897 A > C	Venlafaxine (N06AX16)	C	3	10	42	75	137	0.4 (0.2–0.9)	0.0264	0.0430
*CYP2C19*	rs563052490	chr10:g.96602634 C > A	Esomeprazole (A02BC05)	A	3	2	160	1	1214	15.1(1.4–167.1)	0.0322	0.0310
*CYP2D6*	rs34030679	chr22:g.42610099 T > C	Sertraline (N06AB06)	C	3	11	56	23	273	2.3 (1.1–5.0)	0.0033	0.0076
*CYP2D6*	rs199535154	chr22:g.42524814 A > G	Tolterodine (G04BD07)	G	2 A	2	9	2	72	7.7 (1.0–61.3)	0.0133	0.0133
*CYP2D6*	rs1058172	chr22:g.42523528 C > T	Mirtazapine (N06AX11)	T	2 A	1	13	19	27	0.1 (0.0–0.9)	0.0387	0.0259
*SLC22A2*	rs145259190	chr6:g.160683564 C > T	Metformin (A10BA02)	T	3	6	84	19	666	2.5 (1.0–6.4)	0.0473	0.0473
(c) Insignificant after conditional analysis with previously reported association variants												
*AHR*	rs10249788	chr7:g.17338147 C > T	Olanzapine (N05AH03)	T	3	7	9	15	70	NS	0.030	0.051
*CYP2D6*	rs28371703 (rs28588594, rs1080989, rs1065852)	chr22:g.42525821 G > T (chr22:g.42528224 G > A chr22:g.42527793 C > T, chr22:g.42526694 G > A)	Sertraline (N06AB06)	T	3	11	56	100	196	NS	0.006	0.079
*CYP2D6*	rs28371703	chr22:g.42525821 G > T	Fluoxetine (N06AB03)	T	3	13	46	134	228	NS	0.028	0.135
*CYP2D6*	rs1065852 (rs1080989, rs28588594)	chr22:g.42526694 G > A (chr22:g.42527793 C > T, chr22:g.42528224 G > A)	Mirtazapine (N06AX11)	A	2 A	14	53	129	230	NS	0.033	0.139

Star allele—nomenclature for pharmacogenes, discrete star allele represents either a single genetic variant or a haplotype

*ADE* adverse drug effect, *OR* odds ratio, *95% CI* 95% confidence intervals, *LD* linkage disequilibrium, *AA* alternative allele, *NA* not available, *NS* not significant

(a) Significantly replicated results from previously reported variant-drug associations

(b) Significant new pharmacogenomic gene variants (*n* = 1314) in previously reported gene-drug associations

(c) Variants that became insignificant after correction with previously reported variants in a gene-drug association

Following from this validation approach, we aimed to identify novel variants in pharmacogenes affecting drug response. We examined ADE occurrences among individuals with putative high-impact variants (*n* = 1314) and drug prescriptions that have been associated with the respective genes. We discovered 19 variant associations, most of which were related to CYP genes, which are genetically highly polymorphic [38]. Nine independent signals remained significant after correction for known gene-drug variants (Table 2, Supplementary File 1: Supplementary Figure 3). Four additional associations replicated reported low-evidence (level 3) variant-drug associations (Table 2). To identify novel genetic factors underlying ADEs, we conducted a genome-wide association study (GWAS) among 16,226 subjects considering 43 different drugs that had each been prescribed to at least 1000 Biobank participants (Supplementary Table 3). For each drug, we tested for differences in AFs of 16.5 × 10^6^ single-nucleotide variants (SNVs) among individuals with ADEs compared to controls.

Next, we filtered the genome-wide significant loci (Supplementary Table 8), and based on literature survey, functional and pathway analyses (Supplementary Table 4; sheet 1), we obtained five putative novel SNV-ADE associations (Supplementary File 1: Supplementary Figure 4). To determine the most relevant ADE type, we divided the pooled ADEs into 12 groups based on the physiological pathways and mechanistic properties of the 79 ADE ICD10 codes. We tested the five genotypes against each subset ADE group. Only the subset yielding the lowest *p*-value among the 12 groups (Supplementary Table 1) was used in SNV replications. We replicated the analysis in an independent set of Estonian Biobank samples (634 < *n* < 760) and used Taqman assays for distinct genotyping of the hit SNVs in the five loci. We tested these associations in cases and controls from among individuals who had been prescribed the specific drugs (Supplementary Table 5), using a Bonferroni correction threshold of *p* < 0.01 for the five tests.

**Fig. 2:**
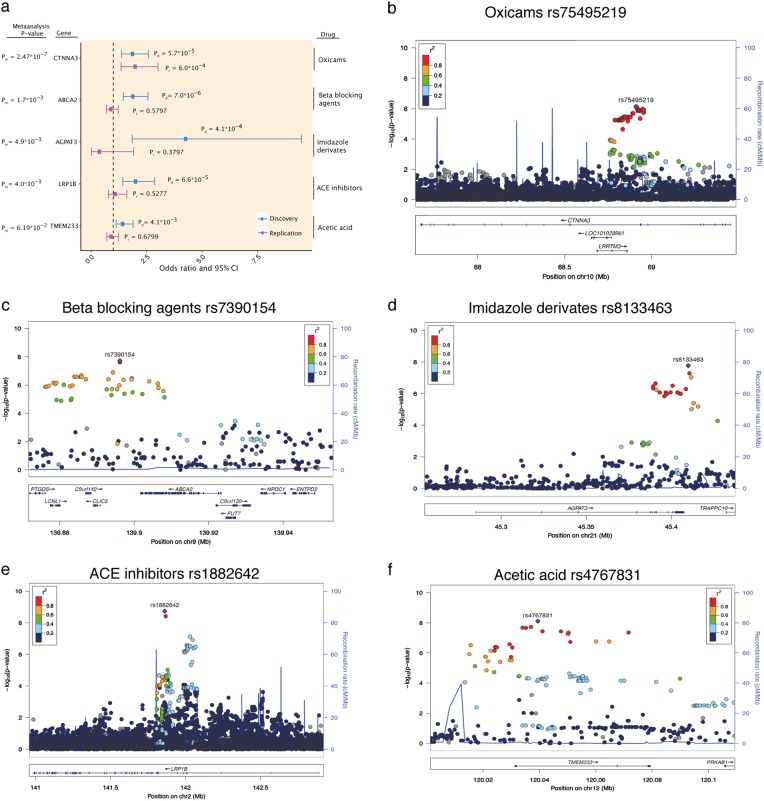
Top five significant findings from genome-wide association analysis (GWAS). **a** Variants selected for replication with odds ratios (squares) and 95% confidence intervals (CI, horizontal lines). Discovery associations with the most significant ADE group are shown in blue and in the replication cohort in purple. The plot is annotated with *p*-values from the discovery (*p*_d_), replication (*p*_r_), and combined meta-analyses (*p*_m_). **b**–**f** Regional association plots for five replicated loci: NM_001127384.2:c.1047 + 29179 T > C (rs75495219); chr11:g.139896164 A > G (rs7390154); NM_020132.4:c.*7617 G > A (rs8133463); NM_018557.2:c.1014-42068 T > C (rs1882642); NM_001136534.1:c.186 + 7589 A > G (rs4767831). Color-coded dots display linkage disequilibrium values for surrounding single-nucleotide variations calculated from the 1000 Genomes Project release of 2012 (EUR population) and human hg19 assembly

**Fig. 3:**
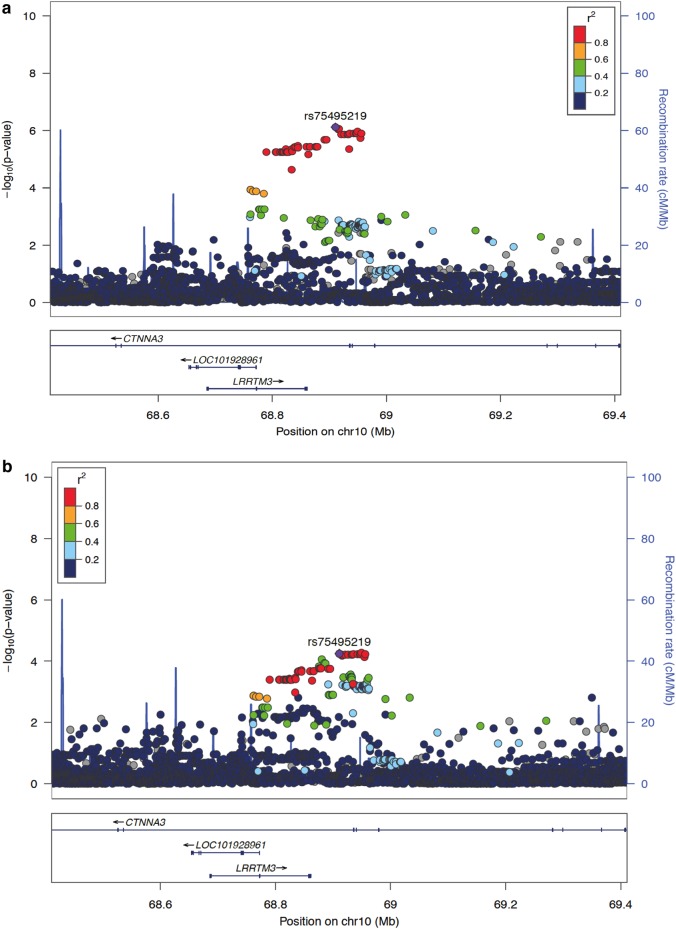
Regional association plots around c.1047 + 29179 T > C (rs75495219) in *CTNNA3 (*NM_001127384.2*)* for adverse drug effects (ADEs) among individuals with oxicam prescriptions. Color-coded dots display linkage disequilibrium values for surrounding single-nucleotide variations calculated from the 1000 Genomes Project release of 2012 (EUR population) and human hg19 assembly. **a** ADEs defined as a set of 79 ICD10 codes. **b** ADEs restricted to a subset of myopathy-related ICD10 codes from **a**

Figure 2 illustrates the ORs and 95% confidence intervals of the five most promising associations from the GWAS in the discovery and replication cohorts. We replicated the association between rs75495219 (replication *p* = 6 × 10^−4^; meta-analysis *p* = 2.47 × 10^−7^) in the seventh intron of the catenin alpha 3 (*CTNNA3*) gene with the occurrence of myopathy-related ADEs among individuals taking oxicams, a class of nonsteroidal anti-inflammatory and anti-rheumatic drugs (Fig. 3). *CTNNA3* has a role in cell adhesion and is mainly expressed in the brain, heart, and muscle cells (Supplementary Table 4; sheet 1; line 46). To rule out a confounding association with inflammation, we tested for a direct association between SNV rs75495219 and 387 unique cases of myopathy/myositis regardless of drug intake in the 16,226 genotyped individuals (logistic regression [LR], *p* = 0.1) (Supplementary File 1: Supplementary Figure 5). A nearby variant (rs61866214) appeared to be significantly associated (*p* = 1.3 × 10^−5^) with myopathy/myositis regardless of drug intake. The *CTNNA3* association remained significant after we adjusted the original rs75495219 association with rs61866214 (*p* = 5.0 × 10^−5^). This result suggests an independent association.

## Discussion

This study is the first to combine EHR and WGS data to investigate ADEs on a population scale. In this proof-of-concept approach, we overlapped three independent sources of data to test the effects of genetic polymorphisms on ADEs among subjects taking specific drugs. Previous studies demonstrated that gene-drug response associations do not require extensive sample sizes for significance due to large effect sizes [39]. In our experience, the intersection of individual medical diagnoses, drug prescriptions, and genotypic alleles are sufficient for population-based inference. Unlike targeted studies, population-based studies identify markers outside specific targeted regions or pathways.

Improvements in quality, quantity, and access to EHR data along with the mass adoption of sequencing-based technologies will provide exciting developments for future studies. The increase in population-specific imputation panels and EHR systems can lead to new associations that use more heterogeneous sources of complex data from various input layers to uncover hypothesis-free relationships and guide research in novel directions. One of the largest ongoing programs for the implementation of pharmacogenomics in the clinic, eMERGE-PGx, is piloting the integration of pharmacogenetic genotypes into the electronic health records and one of their other objectives is to develop a repository of pharmacogenetic variants for further discovery [40]. Their targeted sequencing study of genetic variation in 82 pharmacogenes revealed that 96% of all samples had one or more actionable variants and that 49% of the variants were novel. This highlights the scope of genetic variation in relevant pharmacogenes showing that using sequencing technologies will reveal large numbers of rare variants, and further studies may establish their potential to impact pharmacogenomic traits [41]. With the current study, we highlight the population scale variability in pharmacogenes and demonstrate the possibilities of testing genotype-drug response associations using electronic health records and drug prescription data, thereby providing more resources for validation and further pharmacogenetics discoveries.

We reported that 80% of the variants in the pharmacogenes (n = 64) were rare (MAF < 1%). Rare variant frequencies reported in several other studies highlight the complexities in making between-study estimates comparable. For instance, the data set used in Lakiotaki et al. consisted of 2,504 individuals from 26 different populations and 5 ancestral groups (1000 G Project Phase III) [42]. This study selected 501 PGx variants and identified that the proportion of variants in the lowest reported frequency category, MAF < 5%, varied between 35.8% and 51.2% between-study populations. In contrast, Ingelman-Sundberg et al. aggregated information of 60,000 individuals in the ExAC database from 17 large-scale sequencing projects and reported 98.5% of variants with MAF < 1% [43]. In another study, Mizzi et al. analyzed whole genomes of 482 individuals revealing 408,964 variants in 231 pharmacogenes [44]. Around 58.5% of the variants were singletons and 9.4% were more frequent than AF 20% demonstrating prevalence of rare variants between estimates reported by Lakiotaki et al. and Ingelman-Sundberg et al. Therefore, the reported figures are to be interpreted in consideration of several factors. In larger sample sizes common variants are shared between individuals as rare variation adds to the non-overlapping part [43]. High population stratification increases observed population-specific variants and comparability is also hampered by variable selection of pharmacogenetics variants.

By overlapping the different layers of data, we replicated six and identified nine independent, novel, and putatively high-impact genetic-marker associations with ADEs among groups of individuals stratified by drug prescription (Table 2). Among individuals prescribed metformin we identified a novel association between c.-3775G > A (rs145259190) in a Dnase I hypersensitivity site in the promoter region of *SLC22A2 (NM_003058.3)* (encoding OCT2) and ADEs. This finding aligns with previous studies, which demonstrated effects of genetic variation in OCT2 with decreased renal clearance and increased plasma concentrations of metformin [45, 46], and incentivizes further mechanistic validations.

Three of the other identified associations involved protective effects against ADEs. For example, we observed an association between simvastatin and an upstream variant c.-1023G > A (rs7910642) in *ABCC2 (NM_000392.4)*, which encodes an important efflux pump of endogenous and exogenous compounds [47]. The effect of this SNP on *ABCC2* promoter activity in vitro has been studied before, but no association with *ABCC2* mRNA levels was found [48]. Nevertheless, because *ABCC2* is involved in metabolite efflux [49], and studies have indicated the role of *ABCC2* variants in ADEs or cases of strong reductions in cholesterol levels among patients using simvastatin, this protective effect might be explained by higher elimination of toxic metabolites. Similar assumptions can be made for the association of side-effects from mirtazapine and a non-synonymous variant c.941 G > A (rs1058172) in *CYP2D6* which encodes the primary metabolizer of mirtazapine [50]. Ji et al. previously found this variant to be associated with S-didesmethylcitalopram concentrations, a citalopram metabolite which is converted by *CYP2D6* [51]. This hints at increased levels of *CYP2D6*, which might further explain the protective effect of this variant seen in the current study due to the increased inactivation of mirtazapine by *CYP2D6* [52]. Further investigations are also needed to understand the protective effects found for the c.-91-1825A > T (rs56104268) variant in the *COMT (NM_007310.2)* promoter region among individuals taking venlafaxine. According to previous studies, a missense variant c.322 G > A (rs4680) in *COMT* also appears to affect venlafaxine response despite the small sample sizes of the studies [53, 54].

Previous reports on the relationship between *CTNNA3* variants and drug response described two intronic SNVs, although not at the level of genome-wide significance, which were associated with response to antidepressants resulting in treatment-emergent suicidal ideation [55, 56]. However, these SNVs (c.1733-17064C > T, c.1281 + 21535 A > G) do not appear to be in linkage disequilibrium (LD) (*R*^2^ < 0.005, Estonian population; *R*^2^ < 0.01, EUR population) with rs75495219 or rs61866214. In addition, none of the significant intronic SNVs of *CTNNA3* that we studied appear to be in LD with exonic SNVs of *CTNNA3*. To determine causality, we searched for expression quantitative trait loci (eQTL) signals for rs75495219 in different tissues using the GTEx data set but did not find any significant cis-eQTLs. Poor efficacy of meloxicam has been associated with a variant in another catenin, *CTNNB1* [57, 58]. As shown previously, explaining biological insight for ADE-associated noncoding variants remains challenging [5], and the specific pathways leading to the association between *CTNNA3* and the occurrence of myositis need further functional investigation.

In summary, we identified novel and very rare loss-of-function and missense variants in very important pharmacogenes, and investigated several ADE phenotypes using databases of digitalized health records combined with genome-wide testing, replicating several previously documented variant-drug associations and identifying novel independent signals. The discovery of a new relationship between *CTNNA3* and myositis among individuals treated with oxicams warrants further studies of its mechanistic pathways. We conclude that population-based studies have sufficient statistical power to find new associations, and that EHRs could be successfully applied along with genotype information as a methodology for elucidating relationships between drug responses and genetic variation.

## Electronic supplementary material

Supplementary Methods

Supplementary Information

Supplementary Table 1

Supplementary Table 2

Supplementary Table 3

Supplementary Table 4

Supplementary Table 5

Supplementary Table 6

Supplementary Table 7

Supplementary Table 8

Supplementary Legends

## Data Availability

This paper was presented at the European Society of Human Genetics Conference 2017 as a conference talk with interim findings. The presentation’s abstract was published online in The ESHG 2017 Programme Planner at http://www.abstractsonline.com/Plan/ViewAbstract.aspx?sKey=17144fad-1d87-4739-be1d-b2a79df273b0&cKey=7470ab21-e7ce-4fdc-8389-b0102fa5c438. The authors declare that all data supporting the findings of this study are included in this article and its supplementary information files. Any other data are available upon request from the Estonian Genome Center through data release procedures described at https://www.geenivaramu.ee/en/biobank.ee/data-access.
