# Supplementary material for: Genetic variation in the Estonian population: pharmacogenomics study of adverse drug effects using electronic health records
Source: Eur J Hum Genet. 2018 Nov 12;27(3):442–54. doi: 10.1038/s41431-018-0300-6 (PMC6460570; doi:10.1038/s41431-018-0300-6)
Supplement: Supplementary file 1 — Supplementary Methods [file 41431_2018_300_MOESM1_ESM.docx]

**Genetic variation in the Estonian population: pharmacogenomics study of adverse drug effects using electronic health records**

Tõnis Tasa*^1,2^, Kristi Krebs*^2^, Mart Kals^2^, Reedik Mägi^2^, Volker M. Lauschke^3^, Toomas Haller^2^, Tarmo Puurand^4^, Maido Remm^4^, Tõnu Esko^2^, Andres Metspalu^2^, Jaak Vilo^1^ and Lili Milani**^2,5^

^1^Institute of Computer Science, University of Tartu, Tartu, 50409, Estonia

^2^Estonian Genome Center, Institute of Genomics, University of Tartu, Tartu, 51010, Estonia

^3^Department of Physiology and Pharmacology, Section of Pharmacogenetics, Karolinska Institutet, Stockholm, 171 77, Sweden

^4^Department of Bioinformatics, Institute of Molecular and Cell Biology, University of Tartu, Tartu, 51010, Estonia

^5^Science for Life Laboratory, Department of Medical Sciences, Uppsala University, Uppsala, 751 44, Sweden

* - These authors contributed equally to this work

**** -** Corresponding author

**Supplementary Methods**

**WGS variant calling, data processing and filtering.**

DNA samples were prepared by a PCR-free method. Libraries were sequenced on an Illumina HiSeq X Ten (San Diego, CA, USA) by using 150-basepair (bp) paired-end reads to 30× mean coverage with a median insert size of 400 bp ± 25%, as described in detail by Guo et al^1^. Sequenced reads were aligned to the human reference (GRCh37/hg19) by using BWA-MEM v0.7.7^2^. Picard (v1.136, <http://broadinstitute.github.io/picard>) was applied to flag PCR duplicates. Further data processing was done by the Genome Analysis ToolKit (GATK) v3.4 (v3.4-46)^3^. Single sample variants were called with the GATK HaplotypeCaller algorithm. All single gVCF-files were combined and jointly genotyped. We excluded outlier samples based on high contamination (>5%), high proportion of chimeric alignment (>5%), low call rate (<99%), and genotype discordance. We calculated the numbers of all variants, nonreference variants, and heterozygous variants, the ratio of heterozygous to nonreference variants, and the transition/transversion ratio per sample using the PLINK/SEQ v0.10 (<https://atgu.mgh.harvard.edu/plinkseq>) i-stats module and removed samples more extreme than ±3 SD from population mean.

We applied GATK Variant Quality Score Recalibration to filter variants below a truth sensitivity of 99.8%. We excluded from the analysis variants with an inbreeding coefficient less than -0.3, quality by depth less than 2, call rate less than 90%, or Hardy-Weinberg equilibrium test *p*-value less than 1×10^-9^. We also excluded variants from low-complexity regions^4^, tandem repeats, and segmental duplicates. Low-complexity regions were defined based on hg19 assembly extension hs37d5, segmental duplications (GRCh37) based on the Segmental Duplication DB, and tandem repeats based on the Tandem Repeat Finder (GRCh37 assembly).

**Genotyping and imputation**

Additional samples were genotyped at the Core Facility of the Estonian Genome Center by Infinium CoreExome-24 BeadChips (*n* = 6,396), Illumina HumanCNV370-Duo BeadChips (*n* = 2,658), or Illumina HumanOmniExpress Beadchips (*n* = 8,138). Genotypes were phased by using SHAPEIT2^5^ and imputed to the Estonian WGS reference panel by IMPUTE2^6^ with default parameters. Individuals represented on multiple platforms and individuals without basic phenotype information (e.g., age, sex, body mass index [BMI]) were excluded. We used the SNPRelate package (v1.8.0) in R to calculate four individual principal components (PCs) to account for population stratification^7^.

**Electronic health records**

Estonia has a single-payer health insurance system. The Estonian Health Insurance Fund (EHIF) has collected detailed and personalized billing information for all insured citizens since 2000 (<https://www.haigekassa.ee/en/>) in an administrative database. The EHIF covers 95.5% of the population as the only compulsory health insurance in Estonia (99% of the Estonian Biobank cohort).

The following patient and drug prescription information were extracted from the EHRs: date of issue of a drug prescription; Anatomical Therapeutic Chemical (ATC) classification system code for a prescribed drug; classification of the patient’s disease, according to the International Statistical Classification of Diseases and Related Health Problems, 10^th^ edition (ICD10 code); prescription status; and additional purchase date (if available). Additionally, the National Digital Health Record Database (NDHRD) in Estonia provides free-text medical histories starting from 2010.

At recruitment, the data collector (typically, the primary care physician) filled out a thorough questionnaire of ~330 questions and over 1,000 data fields, including self-reported sensitivity to specific drugs.

**Targeted pharmacogenomics variation**

We only selected pharmacogenomics variants in targeted genes with moderate-to-high predicted Sequence Ontology consequence as annotated by VEP (v84; <https://www.ensembl.org/info/docs/tools/vep>). The predicted function of variants was required to be LoF by the Loss-of-Function Transcript Effect Estimator (LOFTEE) plugin of VEP, deleterious by SIFT^8^, damaging by Polyphen^9^, or deleterious by the profile or stability model in the SNPs3D^10^ (dbSNP 128 build) functional effect database. With additional filtering, we removed all variants in the low-complexity, variable tandem segment repeat, and segmental duplication regions. These filters were applied to all pharmacogenes except *CYP2D6*, whose variation was filtered and confirmed by a copy-number variation (CNV) and k-mer–based approach.

**Discovery and filtering of *CYP2D6* CNVs.**

We used the Genome STRiP CNV discovery pipeline (v2.00.1611)^11^ to detect large deletions, large duplications, and multiallelic CNVs for 2,284 deeply sequenced whole genomes. The pipeline was applied in five separate batches of 450 samples each, due to optimal Genome STRiP performance considerations, with the last batch containing 484 samples. We removed 11 samples that had excessive numbers of calls for which the called variants per sample exceeded the median (across all samples) + 3 median absolute deviations, and removed 42 samples that did not pass WGS quality filters. The final comparison set comprized 2,231 individuals.

The union of discovered sites was genotyped with Genome STRiP’s SVGenotyper module in all batches separately and merged. Duplicate calls were removed with standard Genome STRiP duplicate removal settings (site overlap > 50%, logarithm of odds score of genotype concordance at most discordant sample > 0). We excluded CNVs that were shorter than 1 kb and had a call rate of less than 90%. The CNV region with the smallest basepair difference from its 5’ end to the corresponding 5’ end of the *CYP2D6* (hg19) gene was used to estimate the *CYP2D6* CNV.

*CYP2D6* copy numbers were validated with k-mer frequencies. GListMaker software in GenomeTester4 package was used to create 25-mer list for 2,231 deeply sequenced individuals^12^. GListQuery was then used to find 12,269 k-mer frequencies for three regions near the *CYP2D6* locus from each individual sample. We filtered out non-unique k-mers from the human reference genome and calculated the median k-mer frequency from the remaining 242, 531, and 454 k-mer frequencies. *CYP2D6* copy numbers were calculated with GATK by dividing the median k-mer frequency by the median sequencing coverage.

**Validation of known pharmacogenetic associations: minimum sample size.**

The logistic regression model needs sufficient power for estimating 10 parameter coefficients (intercept, BMI, sex, age, four PCs, genotype, genotyping platform), and the ratio of positive to negative ADE cases is approximately one to four. The minimum sample size was inferred from the rule-of-thumb (*10*k/p*) proposed by Peduzzi et al.^13^, where *k* is the number of covariates and *p* is the proportion of positive cases.

**Variant selection for replication: database queries.**

Various layers of information were reviewed for variant selection. This includes the biological pathways of the genes using the GeneNetwork tool^14^, associated drug function and pharmacokinetic-pharmacodynamic information from Drugbank^15^, and summaries of associated genes from GeneCards^16^. Relevant gene pathways (KEGG, Reactome) and biological roles (molecular function, biological process) were analyzed with GeneNetwork with default settings. The GTEx database^17^ was used for queries of tissue expression data.

**GWAS replication dataset**

For each of the associations to be replicated, (“Variant selection for replication”), we selected up to 1,000 participants with prescriptions of drugs under study from among the additional Biobank participants. Cases (participants with ADEs) were matched with controls (participants without ADE diagnosis) by age and sex. The optimal ratio of cases and controls would have been 1:1; however, in situations where there were not enough ADE cases, we chose a greater number of controls to bring the total number of participants to 1,000. Finally, we only selected cases with diagnosed incidence of ADEs that fell into the most significant ADE subgroup that was detected in the discovery cohort (minimum ratio of 1:41).

**Supplementary References**

1 Guo MH, Nandakumar SK, Ulirsch JC *et al.* Comprehensive population-based genome sequencing provides insight into hematopoietic regulatory mechanisms. *Proc Natl Acad Sci U S A* 2017; **114**: E327–E336.

2 Li H, Durbin R. Fast and accurate long-read alignment with Burrows-Wheeler transform. *Bioinforma Oxf Engl* 2010; **26**: 589–595.

3 McKenna A, Hanna M, Banks E *et al.* The Genome Analysis Toolkit: a MapReduce framework for analyzing next-generation DNA sequencing data. *Genome Res* 2010; **20**: 1297–1303.

4 Li H. Towards Better Understanding of Artifacts in Variant Calling from High-Coverage Samples. *Bioinformatics* 2014; **30**: 2843–2851.

5 Delaneau O, Zagury J-F, Marchini J. Improved whole-chromosome phasing for disease and population genetic studies. *Nat Methods* 2012; **10**: 5–6.

6 Howie BN, Donnelly P, Marchini J. A Flexible and Accurate Genotype Imputation Method for the Next Generation of Genome-Wide Association Studies. *PLOS Genet* 2009; **5**: e1000529.

7 Zheng X, Levine D, Shen J, Gogarten S, Laurie C, Weir B. A High-performance Computing Toolset for Relatedness and Principal Component Analysis of SNP Data. *Bioinformatics* 2012; **28**: 3326–3328.

8 Kumar P, Henikoff S, Ng PC. Predicting the effects of coding non-synonymous variants on protein function using the SIFT algorithm. *Nat Protoc* 2009; **4**: 1073–1081.

9 Adzhubei I, Jordan DM, Sunyaev SR. Predicting functional effect of human missense mutations using PolyPhen-2. *Curr Protoc Hum Genet* 2013; **Chapter 7**: Unit7.20.

10 Yue P, Melamud E, Moult J. SNPs3D: candidate gene and SNP selection for association studies. *BMC Bioinformatics* 2006; **7**: 166.

11 Handsaker RE, Doren V Van, Berman JR *et al.* Large multiallelic copy number variations in humans. *Nat Publ Group* 2015; **47**: 296–303.

12 Kaplinski L, Lepamets M, Remm M. GenomeTester4: a toolkit for performing basic set operations - union, intersection and complement on k-mer lists. *GigaScience* 2015; **4**: 58.

13 Peduzzi P, Concato J, Kemper E, Holford TR, Feinstein AR. A simulation study of the number of events per variable in logistic regression analysis. *J Clin Epidemiol* 1996; **49**: 1373–9.

14 Sloan Z, Arends D, Broman KW *et al.* GeneNetwork: framework for web-based genetics. *J Open Source Softw* 2016; **1**.

15 Law V, Knox C, Djoumbou Y *et al.* DrugBank 4.0: shedding new light on drug metabolism. *Nucleic Acids Res* 2014; **42**: D1091-1097.

16 Safran M, Dalah I, Alexander J *et al.* GeneCards Version 3: the human gene integrator. *Database J Biol Databases Curation* 2010; **2010**: baq020.

17 The Genotype-Tissue Expression (GTEx) project. *Nat Genet* 2013; **45**: 580–585.
