# Supplementary material for: Genetic variation in the Estonian population: pharmacogenomics study of adverse drug effects using electronic health records
Source: Eur J Hum Genet. 2018 Nov 12;27(3):442–54. doi: 10.1038/s41431-018-0300-6 (PMC6460570; doi:10.1038/s41431-018-0300-6)
Supplement: Supplementary file 2 — Supplementary Information [file 41431_2018_300_MOESM2_ESM.docx]

**Genetic variation in the Estonian population: pharmacogenomics study of adverse drug effects using electronic health records**

Tõnis Tasa*^1,2^, Kristi Krebs*^2^, Mart Kals^2^, Reedik Mägi^2^, Volker M. Lauschke^3^, Toomas Haller^2^, Tarmo Puurand^4^, Maido Remm^4^, Tõnu Esko^2^, Andres Metspalu^2^, Jaak Vilo^1^ and Lili Milani**^2,5^

^1^Institute of Computer Science, University of Tartu, Tartu, 50409, Estonia

^2^Estonian Genome Center, Institute of Genomics, University of Tartu, Tartu, 51010, Estonia

^3^Department of Physiology and Pharmacology, Section of Pharmacogenetics, Karolinska Institutet, Stockholm, 171 77, Sweden

^4^Department of Bioinformatics, Institute of Molecular and Cell Biology, University of Tartu, Tartu, 51010, Estonia

^5^Science for Life Laboratory, Department of Medical Sciences, Uppsala University, Uppsala, 751 44, Sweden

* - These authors contributed equally to this work

**** -** Corresponding author

**Supplementary File 1**

# SUPPLEMENTARY FIGURES

Supplementary Figure 1. *HLA-B* allele distribution among 2,240 whole-genome sequences for Estonian Biobank participants**.** Minimum threshold for allele representation is minor allele frequency > 0.5%. Each *HLA-B* allele is shown with percentages of heterozygous and homozygous carriers among the total (*n* = 2,240). Numeric annotation denotes the allele frequency of each allele as a percentage.


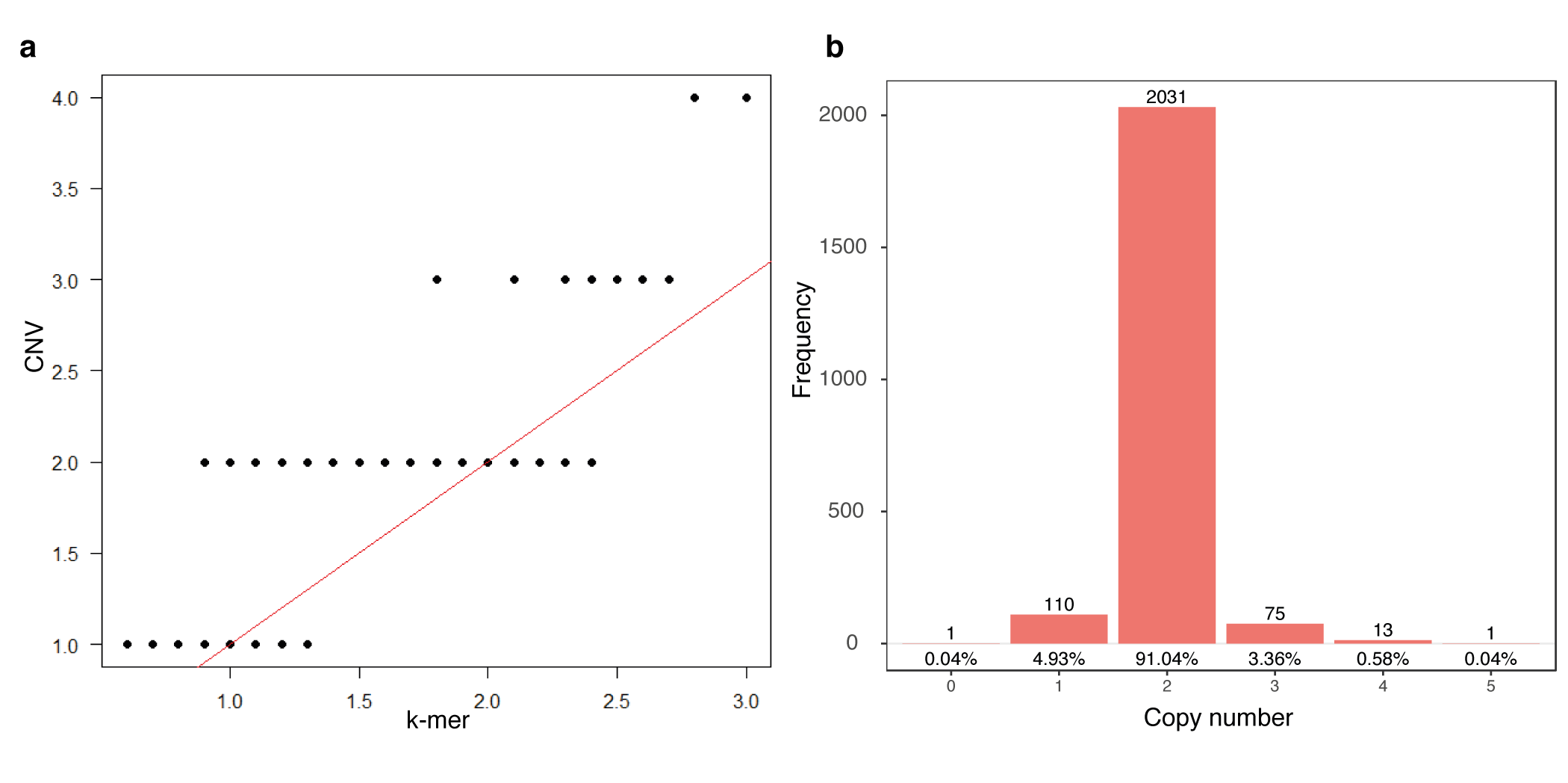


Supplementary Figure 2. Copy-number variation (CNV) and k-mer characterization of *CYP2D6*, called from Estonian whole-genome sequencing data (*n* = 2,231). (a) Frequencies of CYP2D6 CNVs by copy number. (b) Relationship of *CYP2D6* CNV calls to equivalently called k-mers.

**
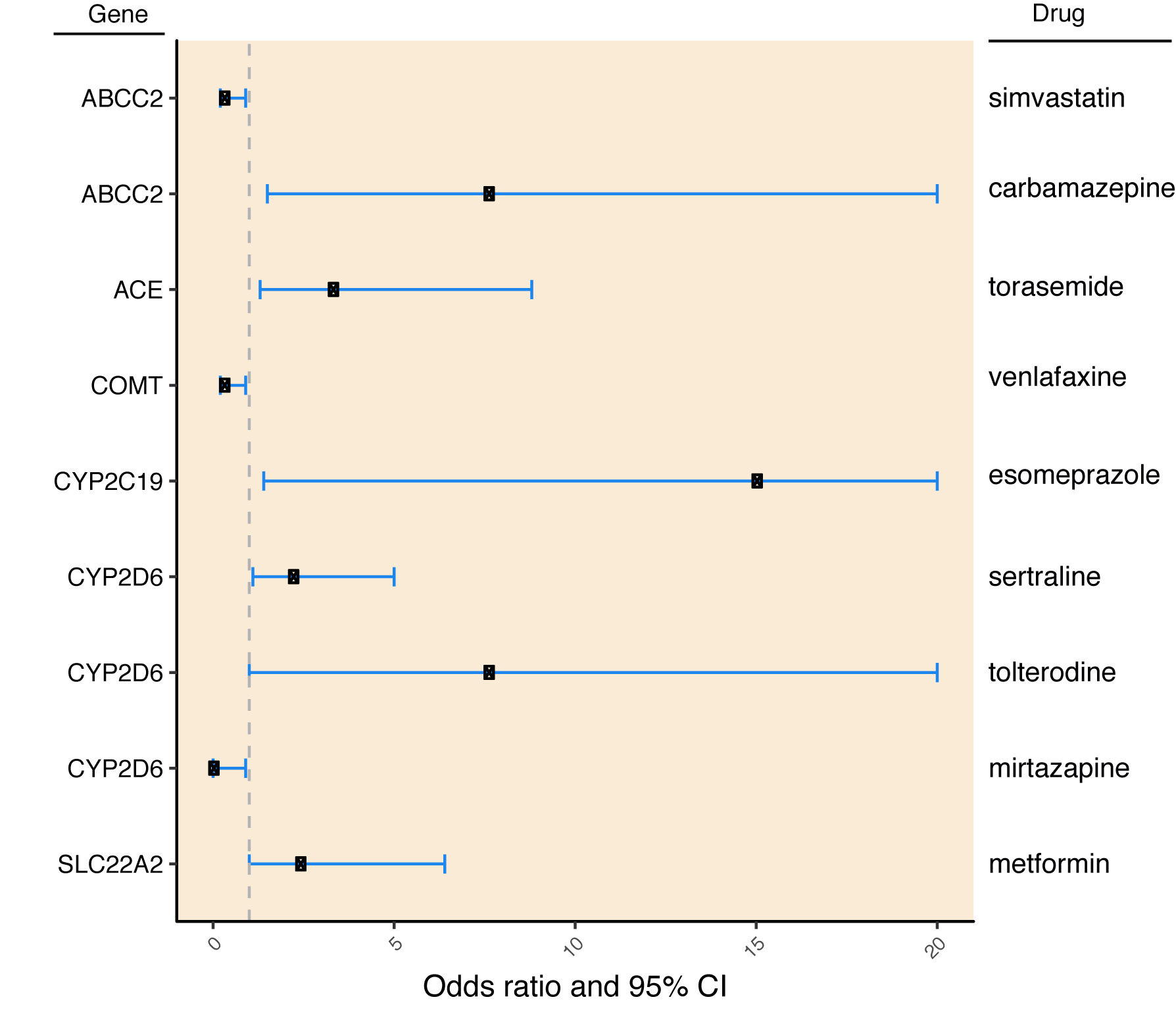
**

Supplementary Figure 3. Nominally significant gene polymorphisms from 64 pharmacogenomic genes (filtered variation, *n* = 1,314) confirming previously reported PharmGKB gene-drug associations. Results represent odds ratios (ORs) with 95% confidence intervals (CIs, horizontal lines).


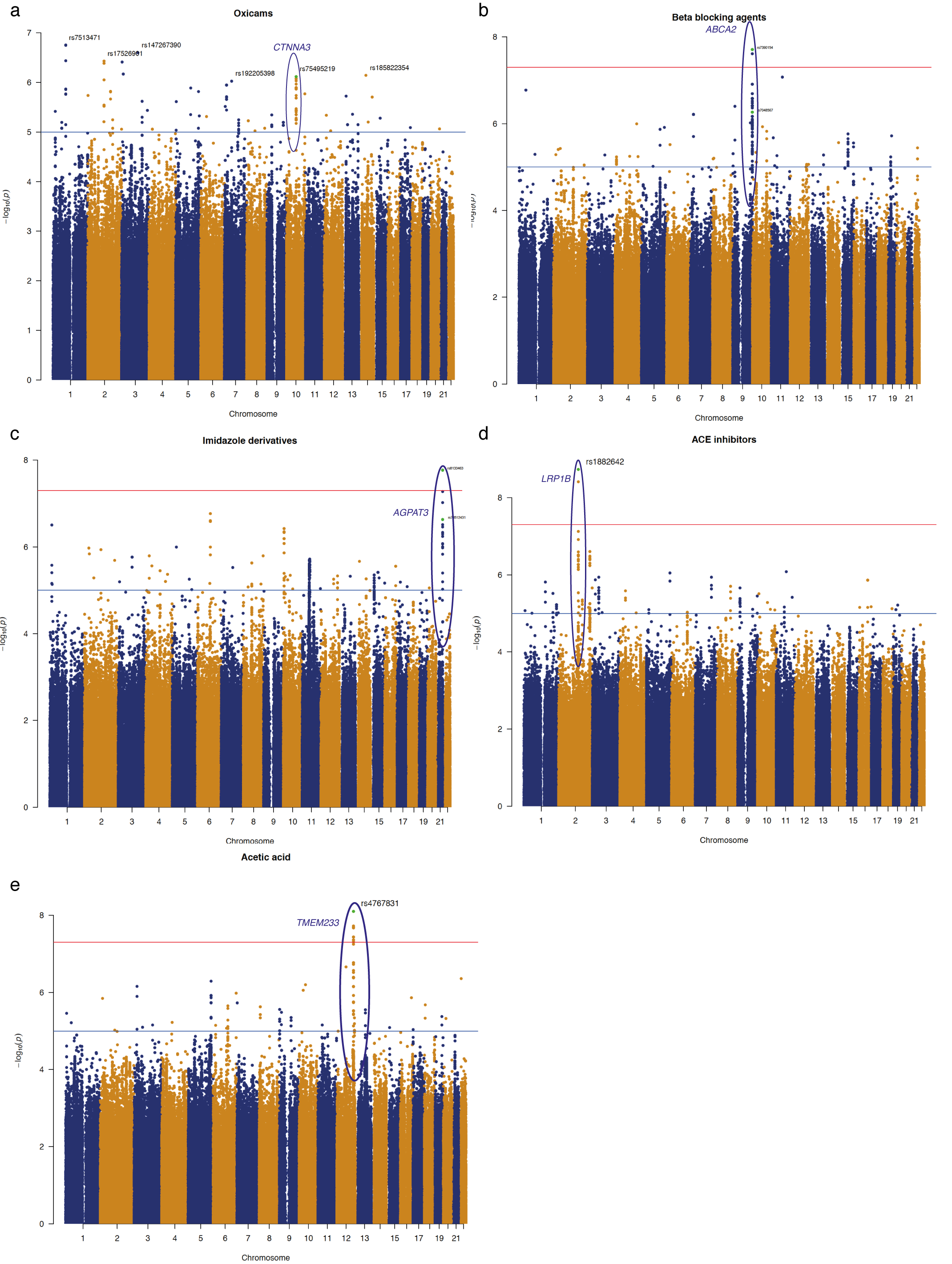


Supplementary Figure 4. Manhattan plots of five replicated variant-drug associations. (a)-(e) Each dot shows log10 *p*-value of a variant at its genomic location. Genome-wide significance (*p*-value = 5×10^−8^) and suggestive threshold (*p*-value = 1×10^-5^) are shown with red and blue lines, respectively. Green dots represent tag-single nucleotide variations (SNVs) with surrounding peaks in blue ellipses annotated with SNV rs number and gene name. Genome reference hg19 (a) chr1:g.91037622G>A (rs7513471), chr14:g.50997920T>A (rs185822354), chr2:g.116631839C>T (rs17526901), chr3:g.116922194T>C (rs147267390), chr7:g.50891096C>T (rs192205398), NM_001127384.2.c.1047+29179T>C / chr10:g.68910896A>G (rs75495219) (b) chr9:g.139896164A>G (rs7390154), chr9:g.139904037A>G (rs7048567) (c) chr21:g.45390737T>C (rs78512431), chr21:g.45409890G>A (rs8133463) (d) chr2:g.141861910A>G (rs1882642) e) chr12:g.120039428A>G (rs4767831).

Supplementary Figure 5. Regional association plots around c.1047+29179T>C (rs75495219) in *CTNNA3 (*NM_001127384.2*)* for myopathy-related adverse drug effects in all individuals. Color-coded dots display linkage disequilibrium values for surrounding single-nucleotide variations calculated using the 1000 Genomes Project release of 2012 (EUR population) and human hg19 assembly.

# SUPPLEMENTARY TABLES

| **SO term** | MAF < 0.5% | MAF 0.5-5 % | MAF > 5% | Total SNPs | **%** |
| --- | --- | --- | --- | --- | --- |
| 3PRIME UTR | 4 | 0 | 1 | 5 | 0.38 |
| 5PRIME UTR | 25 | 4 | 1 | 30 | 2.28 |
| DOWNSTREAM | 10 | 4 | 0 | 14 | 1.07 |
| ESSENTIAL SPLICE SITE | 15 | 1 | 1 | 17 | 1.29 |
| INTRONIC | 195 | 38 | 38 | 271 | 20.6 |
| INTRONIC & WITHIN NON CODING GENE | 1 | 0 | 3 | 4 | 0.30 |
| NON SYNONYMOUS CODING | 470 | 49 | 44 | 563 | 42.9 |
| NON SYNONYMOUS CODING & SPLICE SITE | 17 | 2 | 0 | 19 | 1.45 |
| SPLICE SITE & INTRONIC | 2 | 1 | 0 | 3 | 0.23 |
| STOP GAINED | 23 | 0 | 0 | 23 | 1.75 |
| STOP GAINED & SPLICE SITE | 1 | 0 | 0 | 1 | 0.08 |
| SYNONYMOUS CODING | 11 | 5 | 1 | 17 | 1.29 |
| UPSTREAM | 236 | 36 | 75 | 347 | 26.4 |
| **Total SNPs** | 1010 | 140 | 164 | 1314 | 100 |
|  |  |  |  |  |  |

Extended Table 1. Putative high-impact variation frequencies in pharmacogenetic genes (*n* = 64) by Sequence Ontology (SO) terms and minor allele frequencies (MAFs).

| **Loss-of-function variants in 64 targeted pharmacogenes** | **n** | **%** |
| --- | --- | --- |
| Unique variants | 41 | 3.1 |
| Unique genes with LoF | 25 | 39.1 |
| Unique genes with homozygous LoF | 1 | 1.6 |
| Individuals (n= 2,240) with at least 1 LoF | 727 | 32.5 |
| Individuals (n= 2,240) with at least 1 homozygous LoF | 79 | 3.5 |
| Total LoF variant average in each individual | 0.34 | n/a |
| Essential splice site variants | 17 | 41.5 |
| Stop gained variants | 23 | 56.1 |
| Stop gained & splice site variants | 1 | 2.4 |
| Novel variants | 10 | 24.4 |
| Novel variants found in one allele | 6 | 14.6 |
| Known variants | 31 | 75.6 |
| Known variants found in one allele | 15 | 36.6 |
| Allele count 1 | 21 | 51.2 |
| MAF < 0.05 % | 24 | 58.5 |
| 0.05% < MAF < 1 % | 16 | 39.0 |
| MAF > 5% | 1 | 2.4 |

Extended Table 2. Loss-of-function (LoF) variation characterization in pharmacogenes (*n* = 64) from whole-genome sequences (*n* = 2,240). Variants are characterized by counts, frequencies, functional effects, and minor allele frequencies (MAFs).

| **ICD10 code** | **Diagnosis** | **EHR** | **Self-reported** | **Total** |
| --- | --- | --- | --- | --- |
|  |  | **Number of individuals** | | |
| M60.8 | Other myositis | 226 | 34 | 260 |
| L27.0 | Generalized skin eruption due to drugs and medicaments taken internally | 94 | 10 | 104 |
| M60.9 | Myositis, unspecified | 84 | 17 | 101 |
| L23.3 | Allergic contact dermatitis due to drugs in contact with skin | 83 | 10 | 93 |
| K76.9 | Liver disease, unspecified | 63 | 19 | 82 |
| L27.1 | Localized skin eruption due to drugs and medicaments taken internally | 65 | 3 | 68 |
| J38.4 | Edema of larynx | 40 | 5 | 45 |
| L24.4 | Irritant contact dermatitis due to drugs in contact with skin | 41 | 4 | 45 |
| T42.4 | Poisoning by, adverse effect of and under-dosing of benzodiazepines | 38 | 7 | 45 |
| E03.2 | Hypothyroidism due to medicaments and other exogenous substances | 32 | 8 | 40 |
| T78.3 | Angioneurotic edema | 29 | 6 | 35 |
| R05 | Cough | 0 | 25 | 25 |
| E87.1 | Hypo-osmolality and hyponatremia | 21 | 0 | 21 |
| T45.5 | Poisoning by, adverse effect of and under-dosing of anticoagulants and antithrombotic drugs | 17 | 3 | 20 |
| M10.2 | Drug-induced gout | 15 | 2 | 17 |
| T46.0 | Poisoning by, adverse effect of and under-dosing of cardiac-stimulant glycosides and drugs of similar action | 16 | 1 | 17 |
| T88.7 | Unspecified adverse effect of drug or medicament | 13 | 4 | 17 |
| G62.0 | Drug-induced polyneuropathy | 13 | 2 | 15 |
| T78.2 | Anaphylactic shock, unspecified | 11 | 3 | 14 |
| T42.6 | Poisoning by, adverse effect of and under-dosing of other antiepileptic and sedative-hypnotic drugs | 12 | 1 | 13 |

Extended Table 3. Counts of the top 20 identified adverse drug effects (ADEs) in Estonian Biobank participants. ADEs were categorized by the International Classification of Diseases database 10^th^ revision (ICD10) coding system and collected in the Estonian electronic health records (EHRs). Data were supplemented with information from Biobank questionnaires (self-reported incidences).

# SUPPLEMENTARY TABLE CAPTIONS

Supplementary Table 1. ICD10 codes listed as adverse drug effects, divided into 12 mechanistic pathway groups, with counts of affected individuals based on Estonian electronic health records and self-reported incidents from Estonian Biobank questionnaires.

Supplementary Table 2. List of 64 targeted ADMET-related pharmacogenetically important genes.

Supplementary Table 3. Number of cases (adverse drug effects) and controls (no adverse drug effect) for each of the 43 sets of individuals with specific drug prescriptions evaluated in genome-wide association studies.

Supplementary Table 4. Biological characterization and background information on genome-wide association analysis findings. Sheet “Genome wide associations” presents results for all loci of interest. Sheet “Selected variants” presents results for five loci selected for replication.

Supplementary Table 5. Summary of association analysis results for five single-nucleotide variations (SNVs) selected for replication from genome-wide association analysis. Association analysis tested the effect of genotype (in column “Gene”, “Variant”) on frequencies of adverse drug effects (in columns “ADE”/”No ADE”; “Variant Carriers”/”Non-carriers”) among individuals with prescriptions to a certain drug (in column “ATC code”). Table displays minor allele frequency (MAF) and association results (*p*-values, odds ratios with confidence intervals) of the variants for the discovery and replication sets. Values from the meta-analyses of the discovery and replication sets are presented in the last column.

Supplementary **Table 6.** Loss-of-function variants identified from whole-genome sequences of 2,240 Estonian individuals.

Supplementary Table 7. High-confidence (level of evidence 1A-2B) allele-drug associations from the PharmGKB and respective Estonian cohort test settings and results. Summary table sheet presents an overview of test outcomes. Sheet “all_studied_associations” presents all tested associations. Sheet “Unavailable_for_testing” presents all tests that did not yield a *p*-value. Sheet “Small_sample_size(N<500)” presents all associations that were excluded for the insufficient number of testable individuals. Sheet “Insignificant_results” presents all testable results that yielded insignificant *p*-values.

Supplementary Table 8. Genome-wide association analysis results for adverse drug effect occurrences among individuals with specific drug prescriptions. Analyzed drug (*n* = 43) is given in column “Phenotype/Drug ATC code”. Table presents 63 loci of interest selected on a *p*-value threshold of 1×10^-6^.
