# Supplementary material for: Genetic variation in the Estonian population: pharmacogenomics study of adverse drug effects using electronic health records
Source: Eur J Hum Genet. 2018 Nov 12;27(3):442–54. doi: 10.1038/s41431-018-0300-6 (PMC6460570; doi:10.1038/s41431-018-0300-6)
Supplement: Supplementary file 11 — Supplementary Legends [file 41431_2018_300_MOESM11_ESM.docx]

**Electronic supplementary material**

Supplementary File 1. SupplementaryFile1.docx. Supplementary Figures 1-5, Extended Tables 1-3, captions of Supplementary Tables 1-8

Supplementary Methods. SupplementaryMethods.docx.

Supplementary Table 1. SupplementaryTable1.xlsx. ICD10 codes listed as adverse drug effects

Supplementary Table 2. SupplementaryTable2.xlsx. List of 64 targeted ADMET-related pharmacogenetically important genes

Supplementary Table 3. SupplementaryTable3.xlsx. Number of cases (adverse drug effects) and controls (no adverse drug effect) for each of the 43 sets of individuals with specific drug prescriptions evaluated in genome-wide association studies

Supplementary Table 4. SupplementaryTable4.xlsx. Biological characterization and background information on genome-wide association analysis findings

Supplementary Table 5. SupplementaryTable5.xlsx. Summary of association analysis results for five single-nucleotide variations (SNVs) selected for replication from genome-wide association analysis

Supplementary Table 6. SupplementaryTable6.xlsx. Loss-of-function variants identified from whole-genome sequences of 2,240 Estonian individuals

Supplementary Table 7. SupplementaryTable7.xlsx. High-confidence (level of evidence 1A-2B) allele-drug associations from the PharmGKB and respective Estonian cohort test settings and results

Supplementary Table 8. SupplementaryTable8.xlsx. Genome-wide association analysis results for adverse drug effect occurrences among individuals with specific drug prescriptions
